# Host Gene Signatures Associated with Gastric Cancer–Associated Microbial Taxa: A Descriptive Microbiome–Transcriptome Study

**DOI:** 10.3390/medicina62050799

**Published:** 2026-04-22

**Authors:** Ozgur Albuz, Dilek Pirim, Sevinc Akcay, Tugba Gurkok Tan, Seda Ekici, Sami Akbulut

**Affiliations:** 1 Department of General Surgery, Dr. Abdurrahman Yurtaslan, Ankara Oncology Training and Research Hospital, 06200 Ankara, Türkiye; 2 Department of Translational Medicine, Institute of Health Sciences, Bursa Uludag University, 16059 Bursa, Türkiye; 3 Department of Molecular Biology and Genetics, Faculty of Arts & Science, Bursa Uludag University, 16059 Bursa, Türkiye; 4 Department of Molecular Biology and Genetics, Faculty of Science and Art, Kırşehir Ahi Evran University, 40100 Kirsehir, Türkiye; sevinc.akcay@ahievran.edu.tr; 5 Food and Agriculture Vocational School, Cankiri Karatekin University, 18702 Cankiri, Türkiye; 6 Veterinary Control Central Research Institute, 06220 Ankara, Türkiye; 7 Department of Surgery and Liver Transplantation, Faculty of Medicine, Inonu University, 44280 Malatya, Türkiye; akbulutsami@gmail.com

**Keywords:** gastric cancer, microbiome, dysbiosis, transcriptome, host gene signatures, taxon set enrichment analysis, TCGA

## Abstract

*Background and Objectives*: Gastric cancer remains a leading cause of cancer-related mortality worldwide and develops through complex interactions between environmental factors, microbial dysbiosis, and host molecular pathways. Although *Helicobacter pylori* infection is a well-established risk factor, emerging evidence suggests that broader alterations in the gastric microbiome may also contribute to carcinogenesis. However, the associations between gastric cancer-associated microbial taxa and host gene expression profiles remain insufficiently characterized. This study aimed to identify host gene signatures associated with gastric cancer-related microbial taxa through a descriptive analysis integrating microbiome-derived taxa with transcriptome data. *Materials and Methods*: Microbial taxa associated with gastric cancer were systematically retrieved from the Disbiome database. Taxon set enrichment analysis (TSEA) was performed using the MicrobiomeAnalyst platform to identify host genes associated with gastric cancer-associated taxa. Importantly, TSEA relies on healthy reference data from the Human Microbiome Project and does not establish gastric cancer-specific interactions or causal relationships. Gene expression levels were subsequently evaluated using The Cancer Genome Atlas (TCGA) PanCancer stomach adenocarcinoma (STAD) dataset by comparing tumor and matched normal gastric tissues. Gene interaction network and transcription factor (TF) enrichment analyses were conducted to explore predicted regulatory relationships. *Results*: Among 64 microbial taxa associated with gastric cancer, 43 were reported as elevated. After removing overlapping taxa across studies, 37 elevated and 21 reduced taxa were retained for analysis. TSEA identified 11 host genes associated with gastric cancer-related microbial taxa. Transcriptomic analysis demonstrated significant downregulation of DPP6 and DLG2, while KDM4D, USP34, and VDR were significantly upregulated in gastric cancer tissues compared with normal controls. Network and TF enrichment analyses revealed predicted co-expression and co-localization patterns among these genes, suggesting their potential involvement in immune-related processes, epigenetic regulation, and cellular organization. *Conclusions*: This descriptive study identifies distinct host gene expression signatures associated with gastric cancer-associated microbial dysbiosis. This study is purely associative and hypothesis-generating; no causal or mechanistic inferences are made. TSEA used healthy reference data and therefore does not reflect gastric cancer-specific host–microbe interactions. The findings provide a basis for future hypothesis-driven research but require validation in independent cohorts.

## 1. Introduction

Gastric cancer remains a major global health burden and continues to be a leading contributor to cancer mortality. Based on the GLOBOCAN 2022 estimates, stomach cancer accounted for 968,350 new cases, representing 4.9 percent of all cancers, and 659,853 deaths, corresponding to 6.8 percent of all cancer-related deaths worldwide. These figures place gastric cancer among the top five malignancies for both incidence and mortality [1,2,3]. Despite advances in endoscopic detection, perioperative chemotherapy, and targeted/immunotherapies, outcomes remain suboptimal, largely because many patients present with advanced disease and because gastric cancer exhibits marked biological heterogeneity in terms of histology, anatomical subsite, molecular subtype, and tumor microenvironment diversity. This heterogeneity reflects not only intrinsic genetic alterations but also the influence of environmental and microbial exposures that shape gastric carcinogenesis. Consequently, there is a persistent clinical need to better define the biological interfaces through which these external factors interact with host molecular pathways.

Among these factors, infection with *Helicobacter pylori* (*H. pylori*) represents the most established microbial determinant of gastric cancer and has been classified as a Class I carcinogen by the World Health Organization (WHO) [4]. However, only a subset of infected individuals ultimately develop gastric cancer, indicating that additional microbial, host-related, and environmental components contribute to disease initiation and progression. Increasing evidence suggests that the gastric and gut microbiota beyond *H. pylori* participate in modulating gastric carcinogenesis and may help explain interindividual variability in tumor development [5,6,7].

Recent microbiome studies have consistently reported dysbiosis in patients with gastric cancer, characterized by altered microbial diversity and enrichment of non-Helicobacter taxa compared with healthy controls. These microbial alterations have been proposed to contribute to carcinogenesis through mechanisms including chronic inflammation, production of genotoxic metabolites, disruption of epithelial barrier integrity, and modulation of host immune responses [8,9]. Integrative microbiome–host transcriptome analyses have also been conducted in gastric cancer using multi-omic and correlation-based analytical frameworks to explore associations between microbial taxa and host gene expression patterns [10,11,12].

Although taxonomic shifts in the microbiome have been increasingly described in gastric cancer, the relationships between these microbial alterations and host molecular pathways remain incompletely understood. In particular, systematic investigations describing associations between gastric cancer–associated microbial taxa and host gene expression programs are limited. Host genetic susceptibility also plays a critical role in gastric carcinogenesis. For example, studies have demonstrated a relationship between vitamin D receptor (VDR) signaling and CDH1 regulation, underscoring the importance of epithelial integrity pathways in tumor suppression [13].

In this study, microbial taxa associated with gastric cancer were systematically identified using the Disbiome database, and taxon set enrichment analysis (TSEA) was performed to identify host genes correlated with these taxa. Importantly, the TSEA relies on reference data from healthy individuals (Human Microbiome Project) and therefore does not establish gastric cancer–specific or causal links. The expression patterns of taxa-associated genes were subsequently examined in gastric cancer and matched normal tissues using The Cancer Genome Atlas (TCGA) PanCancer stomach adenocarcinoma (STAD) dataset with multiple-testing correction. Gene interaction networks and transcription factor enrichment analyses were conducted to explore predicted associations among the identified genes. This study is purely descriptive and hypothesis-generating; no causal or mechanistic inferences are made. The analysis compares two independent data sources: literature-derived gastric cancer–associated microbial taxa and TCGA-derived gastric cancer gene expression.

## 2. Materials and Methods

### 2.1. Retrieval of Gastric Cancer–Associated Microbial Taxa

The Disbiome database (https://disbiome.ugent.be, accessed on 15 September 2025) was systematically queried to identify bacterial taxa reported to be associated with gastric cancer [14]. The search term “gastric carcinoma” was used as the primary disease keyword, and filtering was restricted to human studies. Studies lacking explicit case–control comparison, unclear sampling origin, or insufficient methodological detail were excluded. Study characteristics, including sample type and direction of association (increased or decreased abundance), were manually reviewed to ensure consistency. A total of eight eligible studies were retained, comprising analyses of gastric wash, tissue biopsy, feces, and blood samples [15,16,17,18,19,20,21,22].

### 2.2. Taxon Set Enrichment Analysis

Taxon sets were imported into the MicrobiomeAnalyst platform (https://microbiomeanalyst.ca, accessed on 30 September 2025) for taxon set enrichment analysis using the TSEA (Taxon Set Enrichment Analysis) module, which examines genes correlated with microbial taxa and associations with host-intrinsic factors. Gene-microbial taxa associations were identified by integrating data from the Human Microbiome Project Data Coordination Center (dbGaP accession: phs000228), which were analyzed using the Host–Microbiome Interaction Identification (HOMINID) pipeline. The TSEA reference data are derived from healthy individuals (Human Microbiome Project) and therefore do not represent gastric cancer–specific interactions. A total of 53 microbial taxa were analyzed in the TSEA module for functional enrichment analysis. Functional enrichments of taxon sets are calculated by using hypergeometric tests [23]. Given the relatively small number of taxon sets included in the enrichment analysis, a *p*-value < 0.05 was considered statistically significant, and false discovery rate (FDR) correction was not applied. Genes associated with taxon sets meeting this nominal significance threshold were subsequently selected for downstream analyses to investigate their potential involvement in cancer.

### 2.3. Expression Analysis of All Taxa Genes in PanCancer Data

We next examined the expression of the TSEA-identified genes in the independent TCGA STAD dataset. This expression analysis is descriptive and independent of the TSEA. Gene expression data were obtained from the Cancer Genome Atlas (TCGA) PanCancer cohort for stomach adenocarcinoma (STAD). Data were accessed via the ENCORI bioinformatics platform (https://rnasysu.com/encori/panCancer.php, accessed on 10 October 2025). The ENCORI platform provides level 3 normalized RNA sequencing data (RSEM normalized, log2-transformed) for 415 STAD samples, including 375 primary tumor samples and 32 matched adjacent normal gastric tissue samples. All 11 TSEA-identified genes (CTD-2127H9.1/OSMR-AS1, STAB1, C2orf84, DPP6, KDM4D, P2RX7, RXFP2, USP34, UTS2R, VDR, and DLG2) were included in the analysis. Their expression levels were examined in gastric cancer tissues and matched normal gastric tissues.

Normalized expression values were used for all downstream analyses. Differential expression analysis was performed to compare gastric cancer tissues with matched normal tissues for all taxa genes. The fold change was calculated as the ratio of gene expression in cancer samples to that in normal samples. Statistical significance was assessed using two-sided Student’s t-tests as implemented within the ENCORI platform, generating *p*-values for each gene. False discovery rate (FDR) correction was applied to adjust for multiple testing, and genes with an FDR < 0.05 were considered statistically significant.

### 2.4. Functional Network Analysis Using GeneMANIA

The 10 host genes identified by taxon set enrichment analysis were further investigated using the GeneMANIA platform (https://genemania.org) under the Homo sapiens organism setting. Functional associations among genes were explored based on co-expression and co-localization data. The resulting network was visualized to identify potential biological modules and shared regulatory patterns. Network figures were exported for inclusion in the manuscript.

### 2.5. Transcription Factor Enrichment and Network Construction

To identify potential transcription factors (TFs) associated with taxa, enrichment analysis was performed using the ChEA3 platform (https://maayanlab.cloud/chea3, accessed on 14 April 2026). The predicted 10 genes (one of which was lncRNA and therefore excluded from TF enrichment analysis) identified through taxon set enrichment analysis were used as input, and integrated meanRank scores were retrieved. The top-ranked TFs were selected based on ChEA3 integrated ranking results. Predicted TF–gene relationships were extracted from the overlapping gene information provided by ChEA3 outputs. A regulatory interaction network was constructed using R (version 4.5.1) and the igraph package.

## 3. Results

### 3.1. Taxon Sets Associated with Gastric Cancer

The results of 8 studies were extracted, revealing 64 taxa associated with gastric cancer (Supplementary Table S1). Among these 64 taxa, 43 were elevated, and 6 were associated with more than one study. After removing duplicate reports across studies, 37 taxa were identified as elevated and included in our study. Additionally, 21 taxa were identified as reduced in gastric cancer cases (Table 1). Of note, 5 taxa (Neisseria, Streptococcus, Acinetobacter, Bacteroides, Prevotella) were observed to be both elevated and reduced in gastric cancer cases. Therefore, to avoid double-counting in downstream analysis, these overlapping taxa were counted only once, resulting in a final set of 53 unique taxa analyzed in TSEA.

### 3.2. Functional Enrichment Analysis of Taxon Sets

We exported all taxa to MicrobiomeAnalyst for functional analysis, which revealed 11 significant genes [CTD-2127H9.1 (OSMR-AS1), STAB1, C2orf84, DPP6, KDM4D, P2RX7, RXFP2, USP34, UTS2R, VDR, DLG2] associated with gastric cancer-associated taxon sets.

### 3.3. Expression Analysis of All Taxa Genes in PanCancer Data

**Figure 1:**
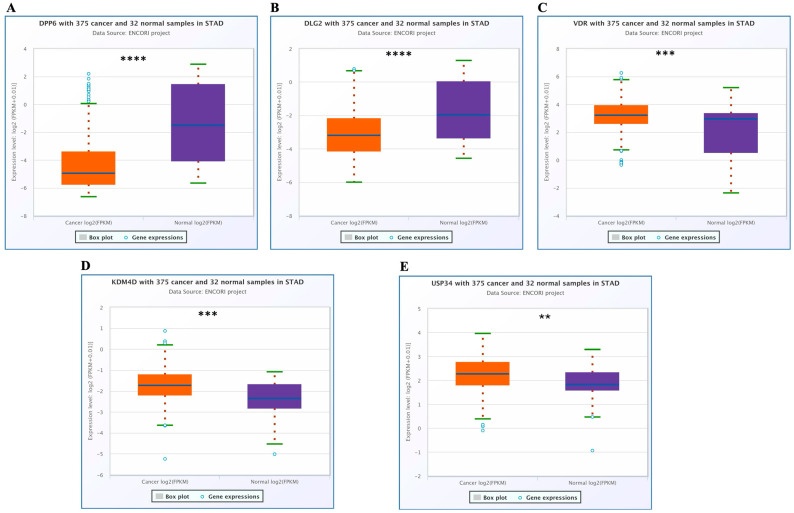
Differential expression of taxa-related genes in gastric cancer (STAD). Boxplots show log_2_-transformed normalized expression values in gastric cancer tissues and matched normal gastric tissues. Panels (**A**–**E**) display the five genes that reached statistical significance after false discovery rate (FDR) correction (FDR < 0.05): (**A**) DPP6, (**B**) DLG2, (**C**) VDR, (**D**) KDM4D, (**E**) USP34. Significance levels (FDR-corrected): ** *p* < 0.01; *** *p* < 0.001; **** *p* < 0.0001. Exact *p*-values and FDR values are provided in Table 2. Gene expression data were obtained from the TCGA PanCancer STAD cohort using the ENCORI platform. Statistical analyses were performed with multiple-testing adjustment using the false discovery rate (FDR).

Differential expression analysis of taxa-related genes was conducted using TCGA PanCancer data to compare gastric cancer (STAD) (*n* = 375) tissues with matched normal gastric tissues (*n* = 32). The expression profiles and statistical outcomes for all 11 genes identified by TSEA are summarized in Table 2. Figure 1 displays boxplots for the five genes that reached statistical significance after FDR correction (FDR < 0.05).

As shown in Table 2 and Figure 1A,B, DPP6 and DLG2 exhibited marked reductions in expression in tumor samples (*p* = 2.3 × 10^−14^ and *p* = 2.5 × 10^−7^), with DPP6 demonstrating the greatest decrease among the analyzed genes. In contrast, VDR expression was modestly but significantly higher in gastric cancer tissues compared with normal controls (*p* = 8.0 × 10^−5^) (Figure 1C). In contrast, KDM4D and USP34 were significantly upregulated in gastric cancer tissues relative to normal samples (*p* = 1.4 × 10^−5^ and *p* = 0.0026, respectively; Table 2; Figure 1D,E).

The lncRNA CTD-2127H9.1 (OSMR-AS1) showed moderate upregulation in tumor tissues (*p* = 0.025); however, this did not reach statistical significance after false discovery rate (FDR) correction (FDR = 0.052) (Table 2). This gene is therefore considered not significant in the primary analysis.

The remaining five TSEA-identified genes—STAB1, C2orf84 (FAM228A), P2RX7, RXFP2, and UTS2R—showed no statistically significant differential expression between gastric cancer and normal tissues (all FDR > 0.05; Table 2).

### 3.4. Functional Network Analysis of Taxa-Associated Genes

Functional network analysis using GeneMANIA was performed to explore the associations among the host genes identified by taxon set enrichment analysis. The resulting network did not reveal direct protein–protein interactions but showed that the genes are mainly connected through co-expression and co-localization patterns. These findings suggest that these genes tend to share similar expression profiles and spatial characteristics rather than forming direct molecular complexes.

**Figure 2:**
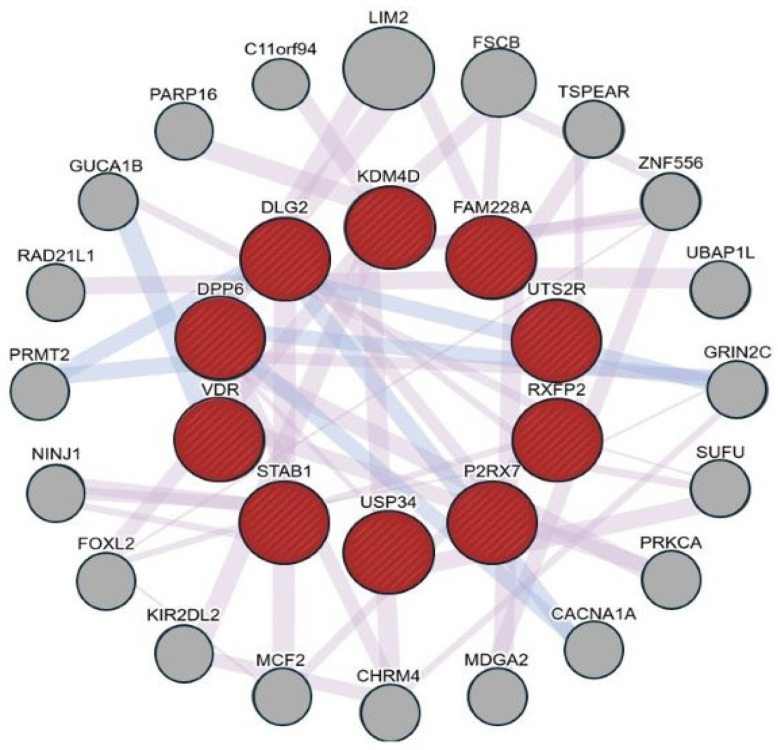
Functional association network of taxa-associated host genes generated using GeneMANIA. Red nodes represent query genes identified through taxon set enrichment analysis (TSEA), while gray nodes indicate additional genes included based on shared co-expression and co-localization patterns. Edges represent co-expression (purple) and co-localization (blue) relationships. No direct protein–protein interactions were identified; therefore, the network reflects computationally inferred associations rather than experimentally validated interactions.

The network analysis grouped the genes into several functional categories, including immune and inflammatory regulation (P2RX7, STAB1, VDR), epigenetic modulation (KDM4D and USP34), and cellular organization and signaling (DLG2, DPP6, RXFP2, and UTS2R) (Figure 2). These groupings are based on computational associations and should be interpreted with caution, as they do not indicate direct functional interactions or causal links. Instead, these findings should be regarded as a hypothesis-generating framework that provides a basis for future experimental validation to further investigate potential host–microbiota relationships in gastric cancer.

### 3.5. TF–Gene Regulatory Network

**Figure 3:**
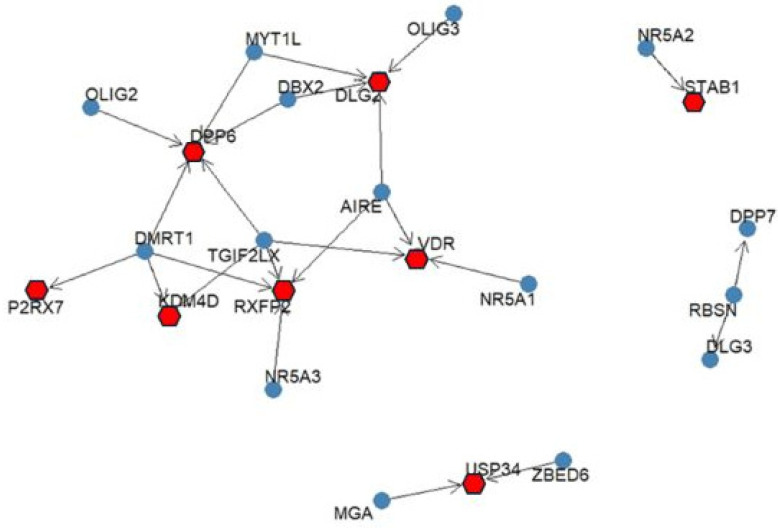
Predicted TF–gene association network derived from ChEA3 enrichment analysis. Blue nodes represent transcription factors, while red nodes indicate taxa-associated host genes. Arrows denote predicted TF–gene associations based on integrated enrichment datasets. The network reflects computationally inferred relationships and does not represent experimentally validated regulatory interactions or causal relationships.

To identify potential upstream regulators of the taxa-associated host genes, TF enrichment analysis was performed using the ChEA3. The TF–gene interaction map revealed that several genes are predicted to be associated with shared TFs (Figure 3). For example, DPP6 was associated with multiple regulators, including OLIG2, MYT1L, DBX2, and DMRT1. Similarly, VDR was linked to TFs such as AIRE, NR5A1, and DMRT1, while RXFP2 showed predicted associations with TGIF2LX and NR5A3. These findings suggest potential regulatory relationships based on computational predictions; however, they do not demonstrate direct regulatory interactions and should be interpreted cautiously. Experimental validation is required to confirm these associations.

## 4. Discussion

In the present study, we integrated microbiome–disease association data with functional enrichment and host transcriptomic analyses to explore molecular links between gastric cancer-associated microbial dysbiosis and host gene expression. Rather than limiting the analysis to taxonomic profiling, we investigated host gene signatures associated with microbial taxa and evaluated their expression patterns in TCGA STAD data. This study is purely descriptive and associative; no causal or mechanistic inferences are made.

Among 64 microbial taxa associated with gastric cancer, 43 were reported as elevated, and after removing overlaps, 37 elevated taxa were retained, while 21 were classified as reduced. Elevated taxa included oral- and gastrointestinal-associated genera such as Streptococcus, Fusobacterium, Prevotella, Veillonella, and Porphyromonas, which have been previously reported in gastric and other gastrointestinal malignancies [24,25,26]. Their enrichment supports the concept of microbial translocation and ecological shifts driven by mucosal barrier disruption, altered gastric acidity, and chronic inflammation. In contrast, reduced taxa included short-chain fatty acid-producing genera such as Faecalibacterium, Roseburia, Lachnospira, and Eubacterium rectale. These observations are descriptive and do not imply causation.

The heterogeneity observed across studies—where taxa such as Neisseria, Streptococcus, Acinetobacter, Bacteroides, and Prevotella were reported as both increased and decreased—highlights the complexity of microbiome alterations in gastric cancer. This variability may reflect differences in tumor stage, anatomical location, sampling method, and regional patient characteristics.

Enrichment analysis identified 11 host genes associated with gastric cancer-related taxa, including CTD-2127H9.1 (OSMR-AS1), STAB1, C2orf84, DPP6, KDM4D, P2RX7, RXFP2, USP34, UTS2R, VDR, and DLG2. TCGA analysis demonstrated marked downregulation of DPP6 and DLG2 in tumor tissues, whereas KDM4D, USP34, and VDR were significantly upregulated. By contrast, CTD-2127H9.1 (OSMR-AS1) did not meet the significance threshold after FDR correction (FDR = 0.052) and is therefore considered not significant in this study. The remaining five genes (STAB1, C2orf84, P2RX7, RXFP2, UTS2R) showed no significant differential expression (all FDR > 0.05).

The pronounced downregulation of DPP6 is notable given its reported role in cellular signaling and regulation of proliferation and migration [27]. Similarly, DLG2, a member of the MAGUK family, has been proposed as a tumor suppressor in multiple cancers [28,29,30,31]. Loss of DLG2 expression has been associated with disruption of cell polarity and adhesion, processes linked to local invasion and lymphatic dissemination.

Conversely, upregulation of KDM4D, a histone demethylase involved in chromatin remodeling and DNA damage responses [32,33], has been observed in gastric tumors. Epigenetic alterations have been associated with tumor aggressiveness and therapeutic resistance. Increased expression of USP34, implicated in Wnt/β-catenin signaling, has been observed in association with activation of oncogenic pathways linked to tumor progression [34].

Although TCGA data demonstrated upregulation of VDR, experimental findings have reported reduced VDR expression in metastatic gastric cancer [3]. This apparent discrepancy may reflect stage-specific regulation, as bulk transcriptomic datasets include heterogeneous tumor and stromal compartments. Increased expression does not necessarily imply functional activation, which depends on ligand availability and downstream signaling competence.

Although VDR and USP34 showed statistically significant differential expression after FDR correction, the absolute fold changes were very small (1.09 and 1.29, respectively). The biological relevance of such modest magnitude changes in bulk tumor tissue is uncertain. These small effect sizes may reflect limited clinical significance or could be influenced by stromal or immune cell composition in the bulk RNA-seq data, potentially diluting cell-type-specific expression signals.

Network analysis using GeneMANIA demonstrated predicted co-expression and co-localization patterns among the taxa-associated host genes. While prior studies have largely focused on single inflammatory cascades such as NF-κB or STAT3 activation in *Helicobacter pylori* infection [35,36,37,38], the present findings show predicted associations among multiple genes across immune, epigenetic, and signaling categories. VDR has been described as a mediator linking microbiota and immune regulation [39], and P2RX7 has been implicated in tumor-associated inflammation and responses to bacterial stimuli [1]. The presence of KDM4D within this network is consistent with the hypothesis that microbial signals are correlated with epigenetic remodeling [40]. However, these predicted patterns are derived from computational analyses of healthy reference data and require experimental validation in gastric cancer-specific contexts.

Although the current study is purely descriptive and cannot establish causal relationships, several microbial-derived factors have been reported in the literature to modulate host pathways relevant to the genes identified here, providing hypotheses for future experimental testing. First, short-chain fatty acids (SCFAs), particularly butyrate and propionate produced by Faecalibacterium, Roseburia, and Lachnospira (taxa reduced in gastric cancer patients), act as histone deacetylase (HDAC) inhibitors and have been shown to regulate VDR expression and modulate immune responses [41]. Second, lipopolysaccharide (LPS) from Gram-negative taxa such as Prevotella and Fusobacterium (elevated in gastric cancer) binds to Toll-like receptor 4 (TLR4) and activates NF-κB signaling, which may influence the expression of epigenetic modifiers including KDM4D [42]. Third, Fusobacterium nucleatum produces specific virulence factors: FadA binds to E-cadherin and activates the Wnt/β-catenin pathway, which has been linked to USP34; Fap2 interacts with the TIGIT receptor to modulate immune responses, potentially affecting P2RX7 and VDR signaling [42,43]. 

Network analysis using GeneMANIA indicated that microbiota-associated host genes are primarily linked through co-expression and co-localization patterns, rather than direct protein–protein interactions. This suggests that these genes may share similar expression profiles without necessarily forming direct molecular complexes. While classical research on *H. pylori* has predominantly focused on specific inflammatory cascades such as the NF-κB or STAT3 pathways [37,38,39,40], our results suggest a more distributed pattern of gene co-expression across diverse functional categories. For instance, VDR and P2RX7 have been previously associated with immune-related processes, while KDM4D has been linked to epigenetic regulation in cancer-related contexts [1,41,42]. However, these interpretations are based on existing literature and computational associations rather than direct evidence from the present analysis. Although recent studies have reported associations between intratumoral microbiome composition and clinical outcomes [43], such relationships were not evaluated in this study.

Therefore, these findings should be interpreted cautiously and considered as hypothesis-generating rather than definitive evidence of causal or mechanistic relationships. They provide a computational framework that requires further experimental validation to better understand potential host–microbiome associations in gastric cancer. Importantly, the intratumoral microbiome model described by Wang et al. [41] demonstrated that microbial profiles within resected gastric tumors were directly associated with prognosis, tumor-free margin effectiveness, and responses to adjuvant chemotherapy and immunotherapy. These findings align with our integrative host–gene analysis and emphasize that microbiome-associated molecular signatures may be associated with tumor biology, though further validation is required.

### 4.1. Limitations

This study has several limitations. First, microbial taxa were retrieved from previously published studies curated in the Disbiome database, introducing inter-study heterogeneity related to sampling sites, sequencing platforms, and regional populations. Second, a fundamental limitation is that the TSEA reference data were derived from healthy individuals (Human Microbiome Project), not from gastric cancer patients. Therefore, the identified gene–microbe associations reflect correlations in a healthy population, not cancer-specific interactions. This study is purely descriptive and associative; no causal or mechanistic inferences are made. Third, although all 11 TSEA-identified genes were analyzed, five showed no significant differential expression (FDR > 0.05), and the fold changes for VDR and USP34 were very small (1.09 and 1.29, respectively). The biological relevance of these modest changes is uncertain. Fourth, taxon set enrichment analysis relied on nominal statistical thresholds without FDR correction, which may increase the risk of type I error. Fifth, TCGA transcriptomic data represent bulk tumor sequencing and do not allow cell-type-specific resolution. Gastric tumors contain heterogeneous cell populations, including cancer cells, cancer-associated fibroblasts, immune cells (T cells, macrophages), and endothelial cells. Consequently, the observed expression differences may reflect changes in cell-type composition (e.g., increased immune infiltration) rather than cancer cell-intrinsic transcriptional alterations. This is particularly relevant for genes such as VDR and P2RX7, which are highly expressed in immune and inflammatory cells. Additionally, tumor purity (the proportion of cancer cells relative to stromal and immune cells) varies considerably across TCGA samples and can confound differential expression results. Lower tumor purity may dilute cancer-specific signals, while higher purity may amplify them. Future studies using computational deconvolution methods, single-cell RNA sequencing, or laser-capture microdissection will be necessary to resolve cell-type-specific contributions and account for tumor purity effects. No external cohort, qPCR, or experimental data were used to confirm the findings. Clinicopathological correlations (e.g., tumor stage, survival) were not evaluated. Therefore, all findings should be considered exploratory and hypothesis-generating, requiring validation in independent datasets and experimental models.

### 4.2. Future Perspectives

Future investigations should prioritize multicenter, standardized study designs using matched intratumoral and adjacent normal gastric tissues to reduce methodological heterogeneity and improve reproducibility across populations. Harmonized sampling protocols and simultaneous microbiome–transcriptome profiling from the same tissue specimens will be essential to strengthen biological inference and enable robust host–microbe association analyses.

To address the observational nature of the current study, experimental validation is required to determine whether specific microbial taxa directly modulate the identified host genes (e.g., VDR, KDM4D, USP34, DPP6, DLG2). Several microbial-derived factors provide hypotheses for future testing: short-chain fatty acids (SCFAs) and secondary bile acids can regulate VDR expression; lipopolysaccharide (LPS) from Gram-negative taxa such as Prevotella and Fusobacterium may activate NF-κB signaling and influence epigenetic modifiers including KDM4D; and Fusobacterium adhesin FadA has been shown to upregulate oncogenic and inflammatory pathways in gastric epithelial cells. Patient-derived gastric cancer organoids co-cultured with live microbial taxa or exposed to these metabolites could be used to directly test these hypotheses.

To move toward clinical utility, several specific validation steps are required. First, the gene expression signature (DPP6, DLG2, VDR, KDM4D, USP34) should be validated in pre-operative endoscopic biopsies to determine whether the signature is detectable in small tissue samples obtained during diagnostic gastroscopy. Second, multi-compartment sampling (tumor core, margin, adjacent normal mucosa, and gastric juice) is needed to assess spatial heterogeneity and identify optimal biopsy sites. Third, prospective cohort studies should evaluate whether the signature correlates with clinically meaningful endpoints such as pathologic stage, disease-free survival, and overall survival. Fourth, a standardized qPCR-based assay with predefined cutoffs would be required for reproducible clinical testing. Until these steps are completed, the gene signatures should be considered hypothesis-generating biomarkers rather than ready for clinical deployment.

Integration of intratumoral microbiome sequencing with spatial transcriptomics and single-cell RNA sequencing will further allow compartment-specific resolution of epithelial, stromal, and immune interactions influenced by microbial signals. This is particularly important because bulk tissue RNA-seq, as used in the present study, cannot distinguish whether differential expression of genes such as VDR and P2RX7 reflects cancer cell-intrinsic changes versus altered infiltration of immune or stromal cells. Single-cell approaches will resolve this ambiguity.

Longitudinal cohort studies spanning precancerous lesions to early-stage gastric cancer may clarify temporal microbial dynamics and identify microbiota-associated molecular transitions during carcinogenesis. Finally, experimental validation using patient-derived organoid systems and germ-free or gnotobiotic murine models will be critical to test these hypotheses further and explore the potential of microbiota-associated molecular signatures.

## 5. Conclusions

In conclusion, this descriptive study identifies microbial taxa and associated host gene expression signatures in gastric cancer. The findings highlight candidate genes that are differentially expressed in gastric cancer tissues and are also correlated with gastric cancer–associated microbial taxa in healthy reference data, including DPP6, DLG2, KDM4D, USP34, and VDR. This study is purely associative and hypothesis-generating. The taxon set enrichment analysis relied on healthy reference data (Human Microbiome Project) and therefore does not establish gastric cancer–specific host–microbe interactions. No causal or mechanistic inferences are made. The findings provide a basis for future hypothesis-driven research but require validation in independent cohorts with matched microbiome and transcriptome data.

## Figures and Tables

**Table 1:** Microbial taxa associated with gastric cancer.

Qualitative Outcome	Organism Name
Elevated	*Phyllobacterium*, *Achromobacter*, *Citrobacter*, *Lactobacillus*, *Clostridium*, *Rhodococcus*, ***Neisseria***, *Aggregatibacter*, *Alloprevotella*, *Porphyromonas endodontalisi*, *Streptococcus mitis*, *Streptococcus oralis*, *Streptococcus pneumoniae*, *Peptostreptococcus*, *Streptococcus anginosus*, *Slackia*, *Gemella*, *Fusobacterium*, *Prevotella melaninogenica*, *Propionibacterium acnes*, *Selenomonas*, *Corynebacterium*, ***Prevotella***, *Shigella*, *Klebsiella*, ***Streptococcus***, *Alistipes*, *Veillonella*, *Bifidobacterium*, *Ruminococcaceae*, *Christensenellaceae*, *Parabacteroides*, *Clostridium cluster XVIII*, *Porphyromonas gingivalis*, *Haemophilus parainfluenzae*, ***Acinetobacter***, ***Bacteroides***
Reduced	***Neisseria***, *Helicobacter*, ***Streptococcus***, *Sphingobium yanoikuyae*, *Vogesella*, *Candidatus portiera*, *Comamonadaceae*, ***Acinetobacter***, *Helicobacter pylori*, *Prevotella copri*, ***Prevotella***, *Bacteriodes uniformis*, *Lachnoclostridium*, *Eubacterium rectale*, *Roseburia*, *Lachnospira*, *Faecalibacterium*, ***Bacteroides***, *Sphingomonas*, *Comamonas*, *Pseudomonas stutzeri*

Taxa reported to be reduced and elevated across different studies were highlighted in bold and treated as single to avoid double-counting in downstream analysis. Genus- and species-level entries (e.g., *Helicobacter* vs. *Helicobacter pylori*) are presented separately as reported in the original studies.

**Table 2:** Differential expression of taxa-related genes in gastric cancer (STAD) from the TCGA PanCancer dataset.

Gene	Cancer Expression	Normal Expression	Fold Change	*p*-Value	FDR
VDR	11.57	10.59	1.09	8.0 × 10^−5^	0.00034
DPP6	0.18	1.63	0.11	2.3 × 10^−14^	6.8 × 10^−13^
KDM4D	0.34	0.23	1.52	1.4 × 10^−5^	7.0 × 10^−5^
USP34	5.14	3.99	1.29	0.0026	0.0076
DLG2	0.19	0.55	0.34	2.5 × 10^−7^	1.9 × 10^−6^
CTD-2127H9.1 (OSMR-AS1)-lncRNA	0.36	0.26	1.41	0.025	0.052
STAB1	6.32	5.51	1.15	0.38	0.47
C2orf84 (FAM228A)	0.10	0.10	0.99	0.35	0.44
P2RX7	1.47	1.00	1.48	0.078	0.13
RXFP2	0.04	0.02	2.23	0.14	0.21
UTS2R	0.82	0.61	1.34	0.23	0.31

## Data Availability

The datasets analyzed during the current study are available from the corresponding author on reasonable request.

## Supplementary Materials

The following supporting information can be downloaded at: https://www.mdpi.com/article/10.3390/medicina62050799/s1, Table S1: Detailed characteristics of microbial taxa associated with gastric cancer extracted from the included studies.

## References

[1] Adinolfi E., De Marchi E., Orioli E., Pegoraro A., Di Virgilio F. (2019). Role of the P2X7 receptor in tumor-associated inflammation. Curr. Opin. Pharmacol..

[2] Sung H., Ferlay J., Siegel R.L., Laversanne M., Soerjomataram I., Jemal A., Bray F. (2021). Global Cancer Statistics 2020: GLOBOCAN Estimates of Incidence and Mortality Worldwide for 36 Cancers in 185 Countries. CA Cancer J. Clin..

[3] Yang J., Tan L., Shi R., Hu Q., Gu Y., Du C. (2025). Vitamin D/Vitamin D receptor signaling suppresses gastric cancer metastasis through autophagy-related protein 13/Beclin1-mediated autophagy. Eurasian J. Med. Oncol..

[4] Wizenty J., Sigal M. (2025). *Helicobacter pylori*, microbiota and gastric cancer-principles of microorganism-driven carcinogenesis. Nat. Rev. Gastroenterol. Hepatol..

[5] Chen C.C., Liou J.M., Lee Y.C., Hong T.C., El-Omar E.M., Wu M.S. (2021). The interplay between *Helicobacter pylori* and gastrointestinal microbiota. Gut Microbes.

[6] Guo Y., Zhang Y., Gerhard M., Gao J.J., Mejias-Luque R., Zhang L., Vieth M., Ma J.L., Bajbouj M., Suchanek S. (2020). Effect of *Helicobacter pylori* on gastrointestinal microbiota: A population-based study in Linqu, a high-risk area of gastric cancer. Gut.

[7] Nasr R., Shamseddine A., Mukherji D., Nassar F., Temraz S. (2020). The Crosstalk between Microbiome and Immune Response in Gastric Cancer. Int. J. Mol. Sci..

[8] Mohamed A.S., Bhuju R., Martinez E., Basta M., Deyab A., Mansour C., Tejada D., Deshpande V., Elias S., Nagesh V.K. (2025). The Gut Microbiome’s Impact on the Pathogenesis and Treatment of Gastric Cancer-An Updated Literature Review. Cancers.

[9] Wen J., Lau H.C., Peppelenbosch M., Yu J. (2021). Gastric Microbiota beyond *H. pylori*: An Emerging Critical Character in Gastric Carcinogenesis. Biomedicines.

[10] Doohan D., Rezkitha Y.A.A., Waskito L.A., Vilaichone R.-k., Yamaoka Y., Miftahussurur M. (2021). Integrating microbiome, transcriptome and metabolome data to investigate gastric disease pathogenesis: A concise review. Expert Rev. Mol. Med..

[11] Park C.H., Hong C., Lee A.R., Sung J., Hwang T.H. (2022). Multi-omics reveals microbiome, host gene expression, and immune landscape in gastric carcinogenesis. iScience.

[12] Zhou D., Xiong S., Xiong J., Deng X., Long Q., Li Y. (2023). Integrated analysis of the microbiome and transcriptome in stomach adenocarcinoma. Open Life Sci..

[13] Lopes N., Carvalho J., Durães C., Sousa B., Gomes M., Costa J.L., Oliveira C., Paredes J., Schmitt F. (2012). 1Alpha,25-dihydroxyvitamin D3 induces de novo E-cadherin expression in triple-negative breast cancer cells by CDH1-promoter demethylation. Anticancer Res..

[14] Janssens Y., Nielandt J., Bronselaer A., Debunne N., Verbeke F., Wynendaele E., Van Immerseel F., Vandewynckel Y.P., De Tré G., De Spiegeleer B. (2018). Disbiome database: Linking the microbiome to disease. BMC Microbiol..

[15] Coker O.O., Dai Z., Nie Y., Zhao G., Cao L., Nakatsu G., Wu W.K., Wong S.H., Chen Z., Sung J.J.Y. (2018). Mucosal microbiome dysbiosis in gastric carcinogenesis. Gut.

[16] Dong Z., Chen B., Pan H., Wang D., Liu M., Yang Y., Zou M., Yang J., Xiao K., Zhao R. (2019). Detection of Microbial 16S rRNA Gene in the Serum of Patients with Gastric Cancer. Front. Oncol..

[17] Ferreira R.M., Pereira-Marques J., Pinto-Ribeiro I., Costa J.L., Carneiro F., Machado J.C., Figueiredo C. (2018). Gastric microbial community profiling reveals a dysbiotic cancer-associated microbiota. Gut.

[18] Hu Y.L., Pang W., Huang Y., Zhang Y., Zhang C.J. (2018). The Gastric Microbiome Is Perturbed in Advanced Gastric Adenocarcinoma Identified Through Shotgun Metagenomics. Front. Cell Infect. Microbiol..

[19] Kageyama S., Takeshita T., Takeuchi K., Asakawa M., Matsumi R., Furuta M., Shibata Y., Nagai K., Ikebe M., Morita M. (2019). Characteristics of the Salivary Microbiota in Patients With Various Digestive Tract Cancers. Front. Microbiol..

[20] Liang W., Yang Y., Wang H., Wang H., Yu X., Lu Y., Shen S., Teng L. (2019). Gut microbiota shifts in patients with gastric cancer in perioperative period. Medicine.

[21] Liu X., Shao L., Liu X., Ji F., Mei Y., Cheng Y., Liu F., Yan C., Li L., Ling Z. (2019). Alterations of gastric mucosal microbiota across different stomach microhabitats in a cohort of 276 patients with gastric cancer. EBioMedicine.

[22] Qi Y.F., Sun J.N., Ren L.F., Cao X.L., Dong J.H., Tao K., Guan X.M., Cui Y.N., Su W. (2019). Intestinal Microbiota Is Altered in Patients with Gastric Cancer from Shanxi Province, China. Dig. Dis. Sci..

[23] Dhariwal A., Chong J., Habib S., King I.L., Agellon L.B., Xia J. (2017). MicrobiomeAnalyst: A web-based tool for comprehensive statistical, visual and meta-analysis of microbiome data. Nucleic Acids Res..

[24] Liu C., Ng S.K., Ding Y., Lin Y., Liu W., Wong S.H., Sung J.J., Yu J. (2022). Meta-analysis of mucosal microbiota reveals universal microbial signatures and dysbiosis in gastric carcinogenesis. Oncogene.

[25] Raoul P., Maccauro V., Cintoni M., Scarpellini E., Ianiro G., Gasbarrini A., Mele M.C., Rinninella E. (2024). Microbiota-Gastric Cancer Interactions and the Potential Influence of Nutritional Therapies. Int. J. Mol. Sci..

[26] Vadhwana B., Tarazi M., Boshier P.R., Hanna G.B. (2023). Evaluation of the Oesophagogastric Cancer-Associated Microbiome: A Systematic Review and Quality Assessment. Cancers.

[27] Ding X.Y., Habimana J.D., Li Z.Y. (2025). The role of DPP6 dysregulation in neuropathology: From synaptic regulation to disease mechanisms. Front. Cell. Neurosci..

[28] Keane S., Améen S., Lindlöf A., Ejeskär K. (2020). Low DLG2 gene expression, a link between 11q-deleted and MYCN-amplified neuroblastoma, causes forced cell cycle progression, and predicts poor patient survival. Cell Commun. Signal..

[29] Shao Y.W., Wood G.A., Lu J., Tang Q.L., Liu J., Molyneux S., Chen Y., Fang H., Adissu H., McKee T. (2019). Cross-species genomics identifies DLG2 as a tumor suppressor in osteosarcoma. Oncogene.

[30] Siaw J.T., Javanmardi N., Van den Eynden J., Lind D.E., Fransson S., Martinez-Monleon A., Djos A., Sjöberg R.M., Östensson M., Carén H. (2020). 11q Deletion or ALK Activity Curbs DLG2 Expression to Maintain an Undifferentiated State in Neuroblastoma. Cell Rep..

[31] Zhuang R.J., Bai X.X., Liu W. (2019). MicroRNA-23a depletion promotes apoptosis of ovarian cancer stem cell and inhibits cell migration by targeting DLG2. Cancer Biol. Ther..

[32] Kim T.D., Shin S., Berry W.L., Oh S., Janknecht R. (2012). The JMJD2A demethylase regulates apoptosis and proliferation in colon cancer cells. J. Cell. Biochem..

[33] Yao W., Wang J., Zhu L., Jia X., Xu L., Tian X., Hu S., Wu S., Wei L. (2021). Epigenetic Regulator KDM4D Restricts Tumorigenesis via Modulating SYVN1/HMGB1 Ubiquitination Axis in Esophageal Squamous Cell Carcinoma. Front. Oncol..

[34] Lui T.T., Lacroix C., Ahmed S.M., Goldenberg S.J., Leach C.A., Daulat A.M., Angers S. (2011). The ubiquitin-specific protease USP34 regulates axin stability and Wnt/β-catenin signaling. Mol. Cell. Biol..

[35] Jamal Eddin T.M., Nasr S.M.O., Gupta I., Zayed H., Al Moustafa A.E. (2023). *Helicobacter pylori* and epithelial mesenchymal transition in human gastric cancers: An update of the literature. Heliyon.

[36] Lamb A., Chen L.F. (2013). Role of the *Helicobacter pylori*-induced inflammatory response in the development of gastric cancer. J. Cell. Biochem..

[37] Piao J.Y., Kim S.J., Kim D.H., Park J.H., Park S.A., Han H.J., Na H.K., Yoon K., Lee H.N., Kim N. (2020). *Helicobacter pylori* infection induces STAT3 phosphorylation on Ser727 and autophagy in human gastric epithelial cells and mouse stomach. Sci. Rep..

[38] Wu M., Tian C., Zou Z., Jin M., Liu H. (2024). Gastrointestinal Microbiota in Gastric Cancer: Potential Mechanisms and Clinical Applications-A Literature Review. Cancers.

[39] Clark A., Mach N. (2016). Role of Vitamin D in the Hygiene Hypothesis: The Interplay between Vitamin D, Vitamin D Receptors, Gut Microbiota, and Immune Response. Front. Immunol..

[40] Berry W.L., Janknecht R. (2013). KDM4/JMJD2 histone demethylases: Epigenetic regulators in cancer cells. Cancer Res..

[41] Wang G., Wang H., Ji X., Wang T., Zhang Y., Jiang W., Meng L., Wu H.J., Xing X., Ji J. (2024). Intratumoral microbiome is associated with gastric cancer prognosis and therapy efficacy. Gut Microbes.

[42] Gamble L.A., Grant R.R.C., Samaranayake S.G., Fasaye G.A., Koh C., Korman L., Asif B., Heller T., Hernandez J.M., Blakely A.M. (2023). Decision-making and regret in patients with germline CDH1 variants undergoing prophylactic total gastrectomy. J. Med. Genet..

[43] Zhao Z., Li N., Xu J., Ren J., Yang X., Li X., Yao J., Wu S. (2025). Noncoding RNAs in host–microbiota interaction. Anim. Res. One Health.

